# Chronic Implant-Associated Osteomyelitis of the Distal Humerus: A Case Report of Latissimus Dorsi Pedicled Flap Reconstruction

**DOI:** 10.7759/cureus.114417

**Published:** 2026-08-12

**Authors:** Badamutlang Dympep, Chirag Sharma, Tashi G Khonglah, Bhaskar Borgohain

**Affiliations:** 1 Plastic and Reconstructive Surgery, North Eastern Indira Gandhi Regional Institute of Health and Medical Sciences, Shillong, IND; 2 Orthopaedics and Traumatology, North Eastern Indira Gandhi Regional Institute of Health and Medical Sciences, Shillong, IND

**Keywords:** chronic osteomyelitis, elbow fracture, elbow instability, fracture-related infection, implant-associated infection, pedicled latissimus dorsi myocutaneous flap, soft tissue reconstruction

## Abstract

A man in his 20s developed chronic osteomyelitis following surgical fixation of a compound distal humerus fracture. He presented with pain, discharging sinuses from the operated site, and restricted elbow movement. A staged limb salvage strategy was employed. The first stage involved implant removal, extensive bone and soft tissue debridement with antibiotic cement bead placement, and primary skin closure. Post-surgery, he developed wound dehiscence and infection, not treated by antibiotic therapy. A secondary wound debridement along with negative pressure therapy was done. Three weeks later, once the infection was controlled, reconstruction using a pedicled latissimus dorsi musculocutaneous flap with definitive bony fixation was done. At one-year follow-up, the patient demonstrated satisfactory wound healing, no recurrent infection, and meaningful improvement in pain and function. This case underscores the value of staged, multidisciplinary management in complex post-traumatic elbow infections and establishes the pedicled latissimus dorsi flap as a reliable reconstructive option.

## Introduction

Distal humerus fractures are complex injuries that often require surgical fixation to restore elbow stability and functional movement. Open reduction and internal fixation using dual plating techniques remains the standard method of treatment for displaced intra-articular fractures of the distal humerus [1]. Despite advances in fixation techniques, these injuries continue to be associated with complications such as stiffness, non-union, implant failure, and infection, particularly in patients with open fractures or severe soft tissue injury [2].

Chronic post-traumatic osteomyelitis around the elbow presents a major reconstructive challenge because of the limited soft tissue coverage and the superficial location of the joint [3]. Persistent infection may lead to sinus formation, exposed implants, scarring, joint stiffness, and significant functional limitation. Management in such cases requires not only eradication of infection but also restoration of skeletal stability and durable soft tissue coverage to preserve limb function [4].

The principles of treatment for chronic implant-associated infection include aggressive debridement, removal of infected hardware, culture-directed antibiotic therapy, management of dead space, and timely soft tissue reconstruction [2,5]. In cases where there is extensive soft tissue loss or exposed bone and implants, vascularized muscle flaps play an important role by improving local vascularity, enhancing antibiotic delivery, and providing stable wound coverage [6].

Among the available reconstructive options for elbow defects, the pedicled latissimus dorsi flap remains a reliable and versatile choice because of its robust vascular pedicle, adequate muscle bulk, and wide arc of rotation [7,8]. The flap provides durable soft tissue coverage without the need for microvascular anastomosis and has shown favorable outcomes in complex traumatic and infected elbow wounds [9,10]. Although the pedicled latissimus dorsi flap has been described for complex elbow defects, its use in chronic implant-associated distal humerus osteomyelitis following failed fixation is less commonly reported.

We present a case of chronic implant-associated distal humerus osteomyelitis with soft tissue loss that was successfully managed using staged infection control with pedicled latissimus dorsi flap reconstruction.

## Case presentation

**Figure 1:**
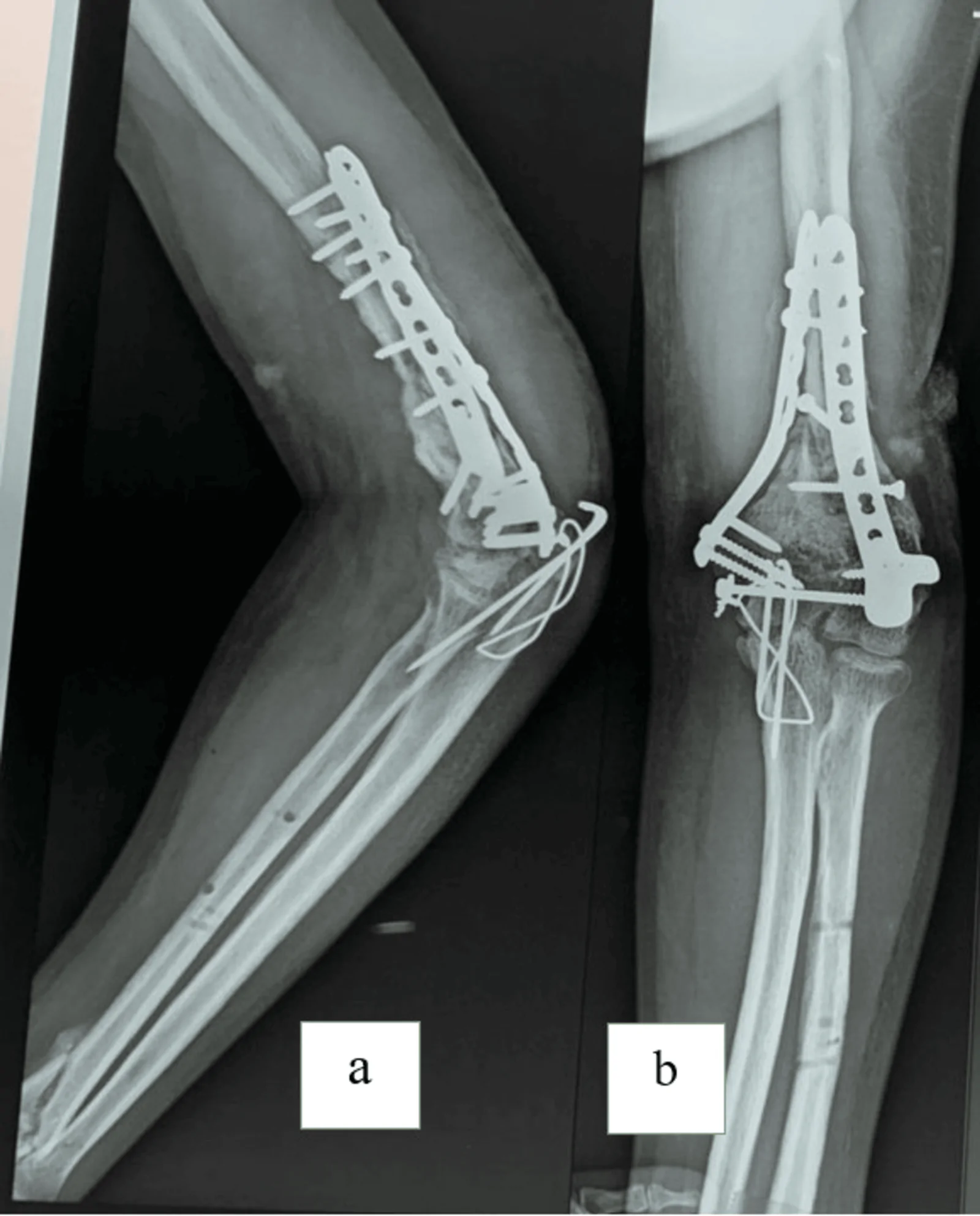
Preoperative X-ray showing distal humerus fracture with implant in situ: (a) lateral view and (b) anteroposterior view

A man in his late 20s with no comorbidities was involved in a road traffic accident in which he sustained distal humerus and distal radius fractures of the left upper limb. He underwent open reduction and internal fixation of the distal humerus fracture using bicondylar plating through an olecranon osteotomy approach with tension band wiring (TBW) of the olecranon (Figure 1).

Post-surgery, the patient developed progressive swelling around the elbow associated with persistent pain and purulent discharge from the operated site. He underwent multiple debridement procedures and antibiotic therapy at several hospitals without improvement.

Six months after the surgery, the patient presented to our center. Clinical examination revealed three actively discharging sinuses around the posterior aspect of the elbow with surrounding induration and scarred soft tissue, with exposed implants and hardware. Elbow movements were painful and significantly restricted, with a range of motion from 30° to 80°. Distal neurovascular status of the limb was preserved.

Laboratory investigations demonstrated elevated inflammatory markers, including erythrocyte sedimentation rate and C-reactive protein levels. Pus culture grew *Escherichia coli*, sensitive to culture-directed antibiotics. The patient's laboratory investigations at presentation are summarized in Table 1. The patient was started on broad-spectrum intravenous (IV) antibiotics.

**Table 1:** Baseline laboratory investigations at presentation demonstrating elevated inflammatory markers consistent with chronic implant-associated infection TLC: total leukocyte count, ESR: erythrocyte sedimentation rate, CRP: C-reactive protein

Investigation	Patient value	Reference range
TLC	13 × 10⁹/L	4-11 × 10⁹/L
ESR	70 mm/hour	0-20 mm/hour
CRP	25 mg/L	0-10 mg/L
Pus culture	Positive for *Escherichia coli*	No growth

**Figure 2:**
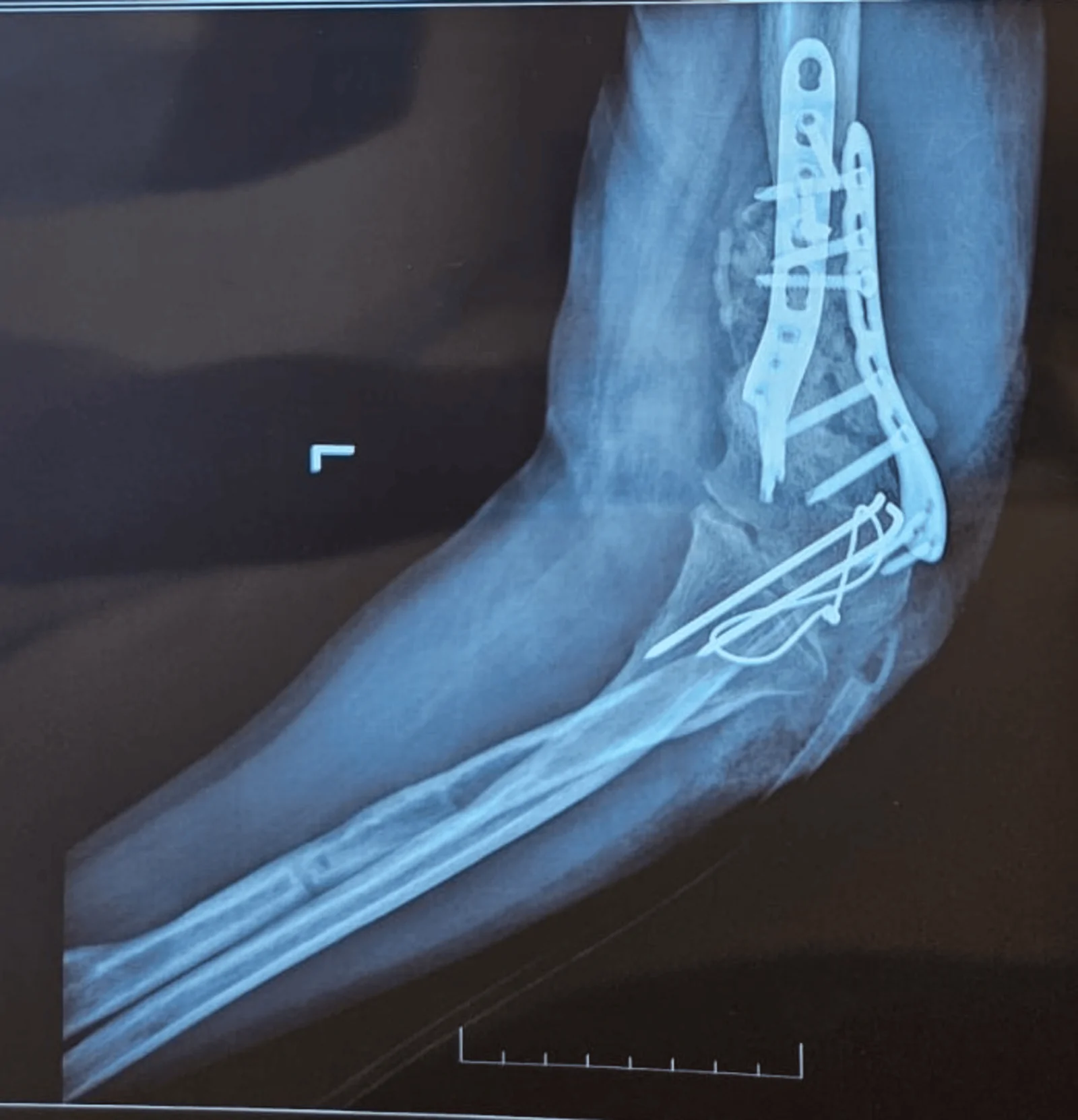
Plain radiographs of the elbow showing retained distal humerus implants with features suggestive of chronic implant-associated infection

Plain radiographs of the elbow showed retained distal humerus implants with features suggestive of chronic implant-associated infection (Figure 2).

Based on the clinical, radiological, and microbiological findings, a diagnosis of chronic implant-associated distal humerus osteomyelitis with associated soft tissue defect was established.

**Figure 3:**
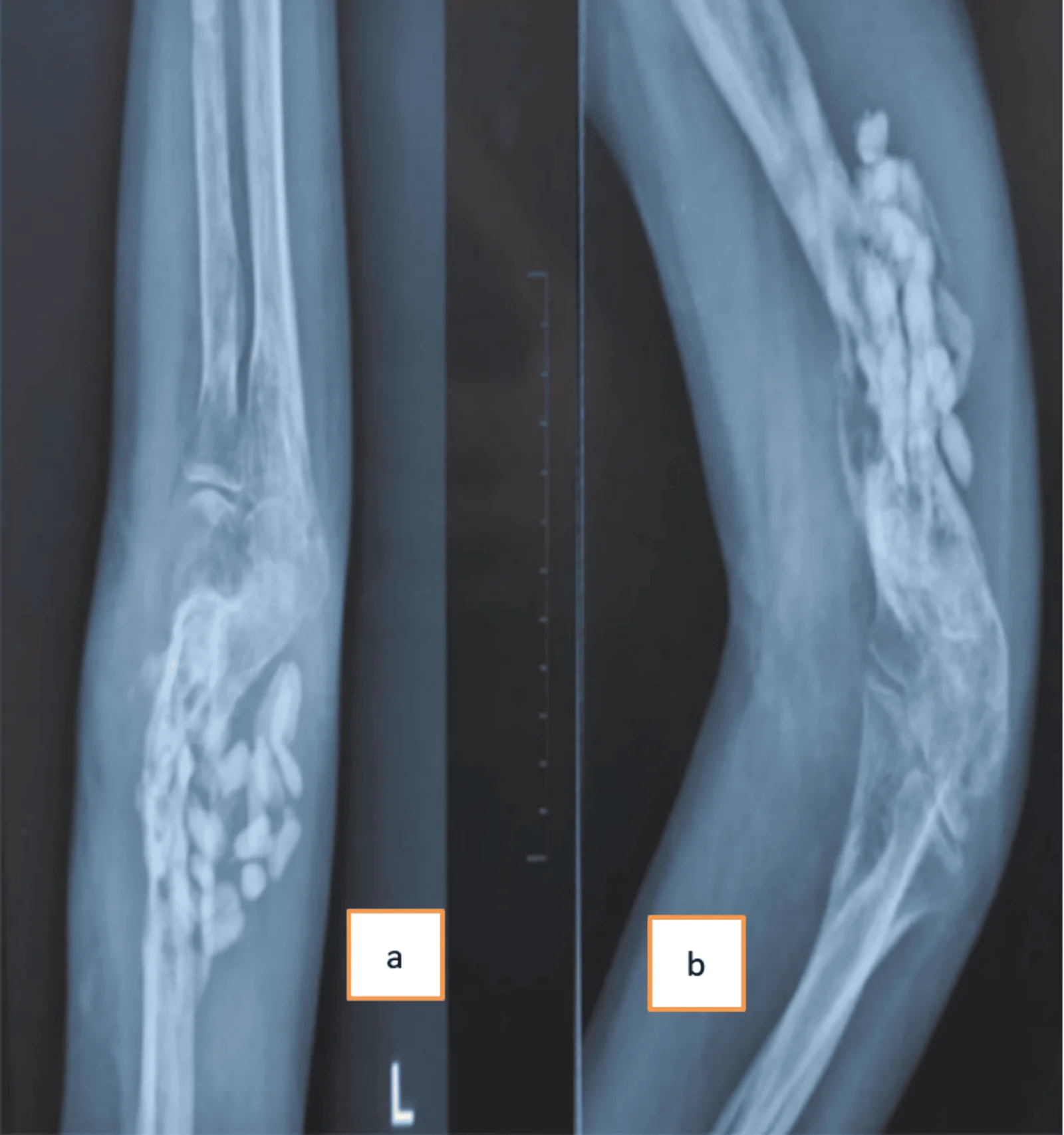
Infection control: implant removed and antibiotic bone cement beads placed

In view of the chronic infection and compromised soft tissue envelope, a staged limb salvage protocol was planned. During the first stage, the previously implanted hardware was removed, and extensive debridement of all necrotic and infected tissue was performed. Antibiotic-impregnated bone cement beads containing gentamicin were placed following copious irrigation (Figure 3).

Postoperatively, the patient developed wound dehiscence; hence, a secondary wound debridement was done, and negative pressure wound therapy was applied over the infected open wound and maintained at -125 mmHg. Culture-sensitive intravenous antibiotics were administered postoperatively.

**Figure 4:**
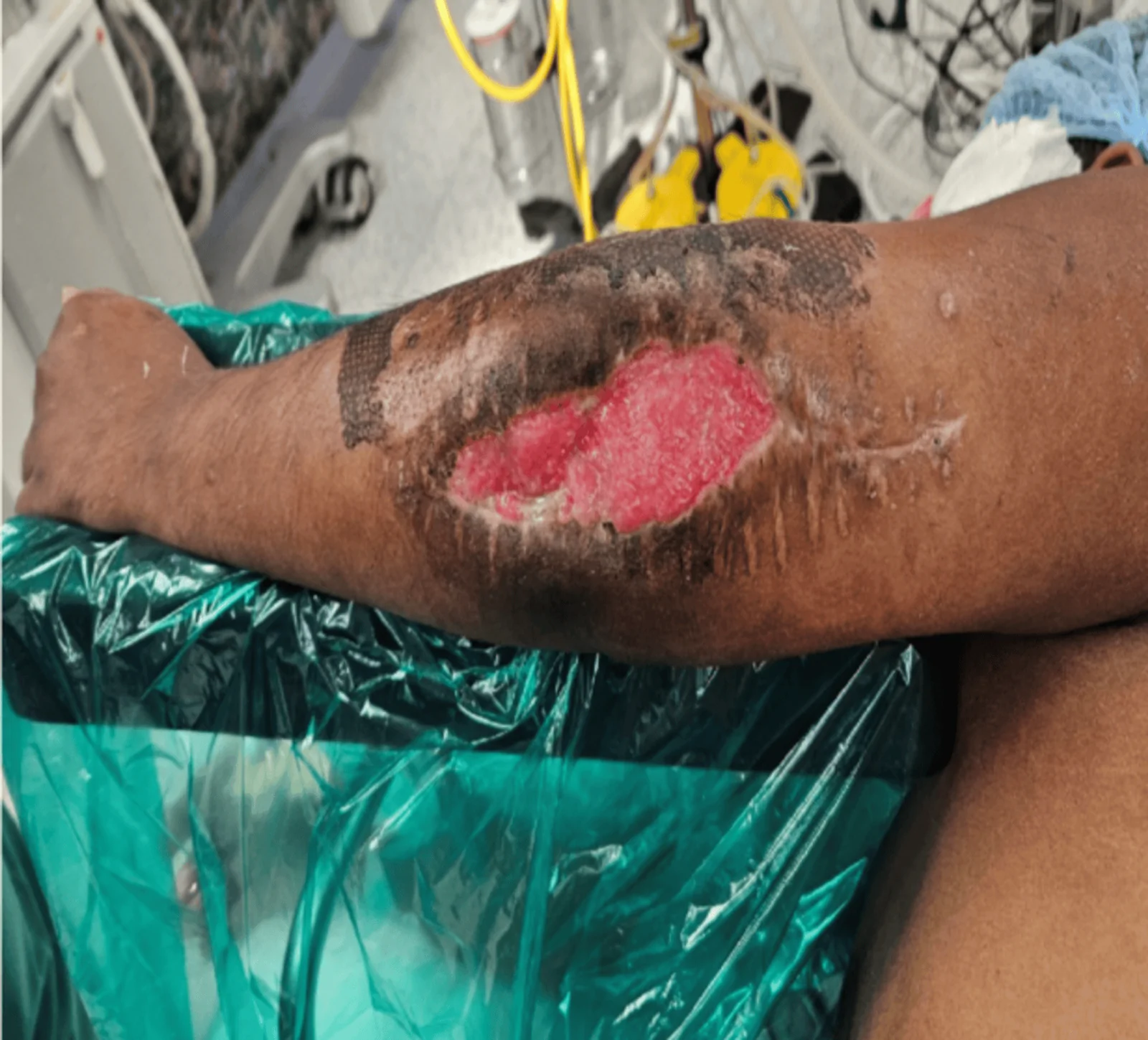
Pre-flap coverage wound status

At three weeks, we achieved satisfactory control of infection and improvement in local wound status (Figure 4). The inflammatory markers ESR and CRP were within normal limits.

**Figure 5:**
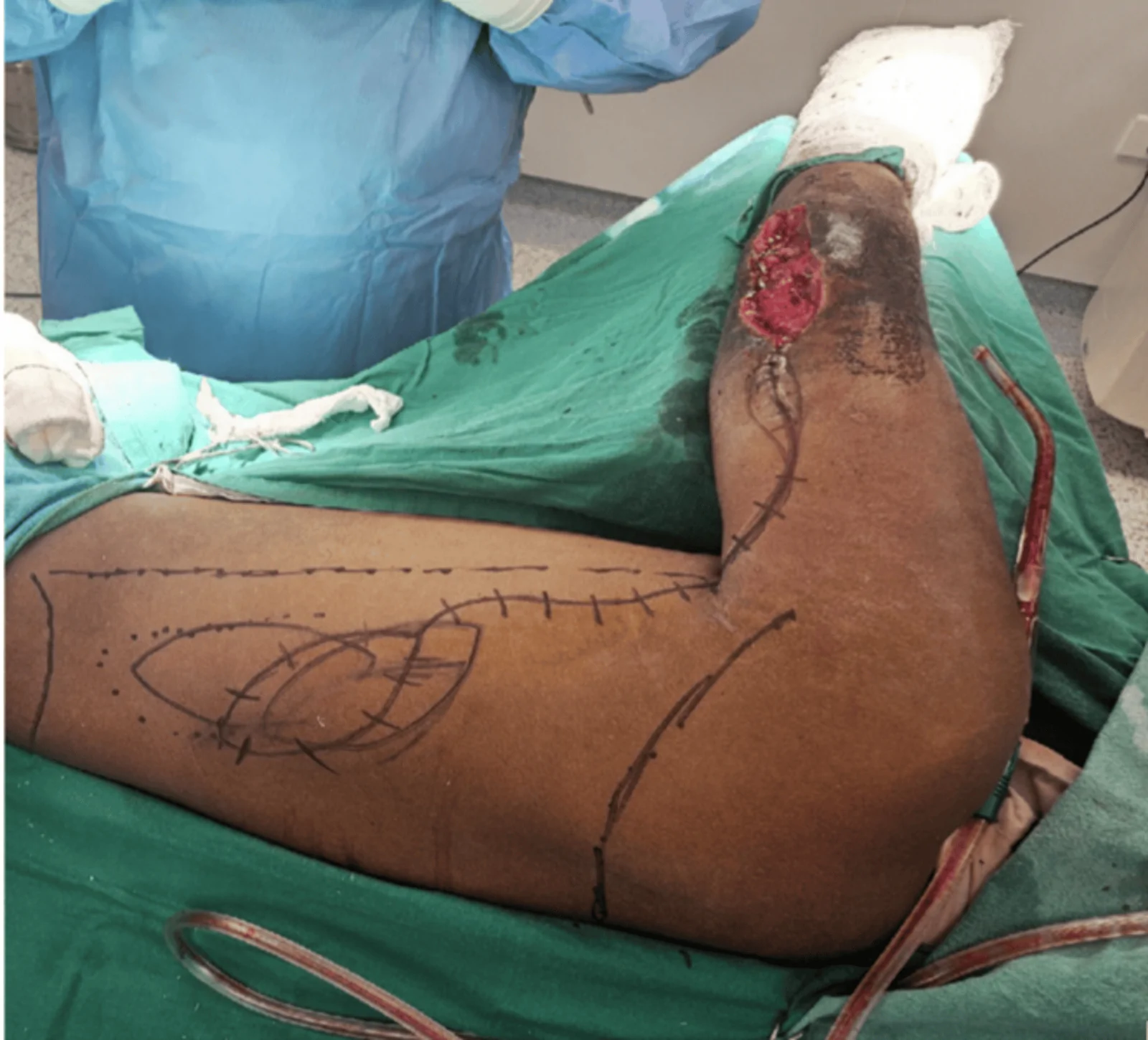
Skin incision markings for latissimus dorsi flap

**Figure 6:**
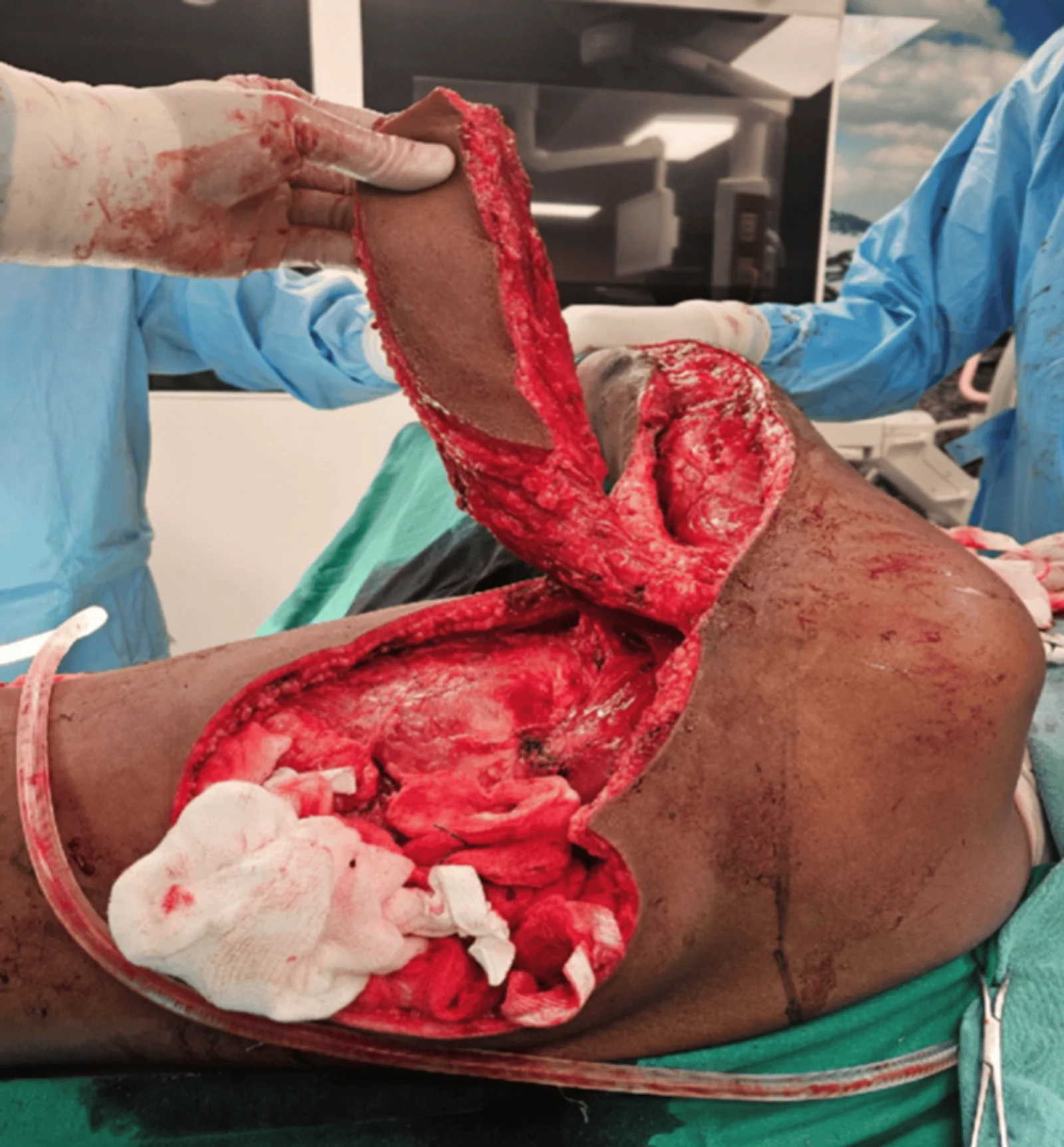
Rotated latissimus dorsi flap

We performed a definitive fracture stabilization to restore alignment of the distal humerus along with soft tissue reconstruction using a pedicled latissimus dorsi musculocutaneous flap. Intraoperative flap markings were made over the latissimus dorsi muscle, after which the flap was elevated on its thoracodorsal vascular pedicle and rotated to cover the elbow defect fully. The flap provided durable, well-vascularized soft tissue coverage over the exposed region and implant (Figure 5 and Figure 6).

**Figure 7:**
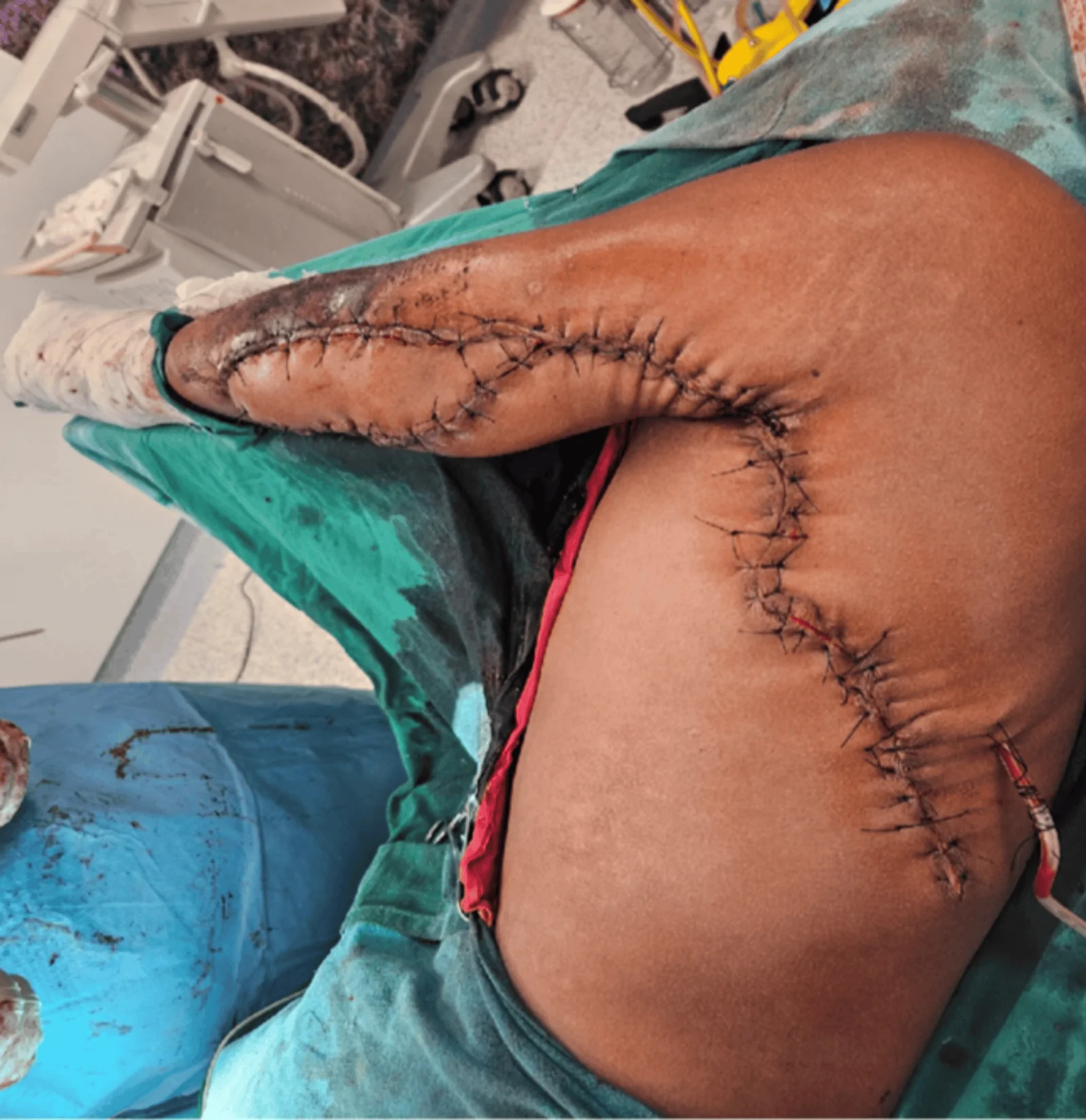
Wound status at the end of surgery

The postoperative period was uneventful, with progressive wound healing and no evidence of recurrent discharge or flap-related complications. At completion of the procedure, stable soft tissue coverage over the elbow was achieved (Figure 7).

**Figure 8:**
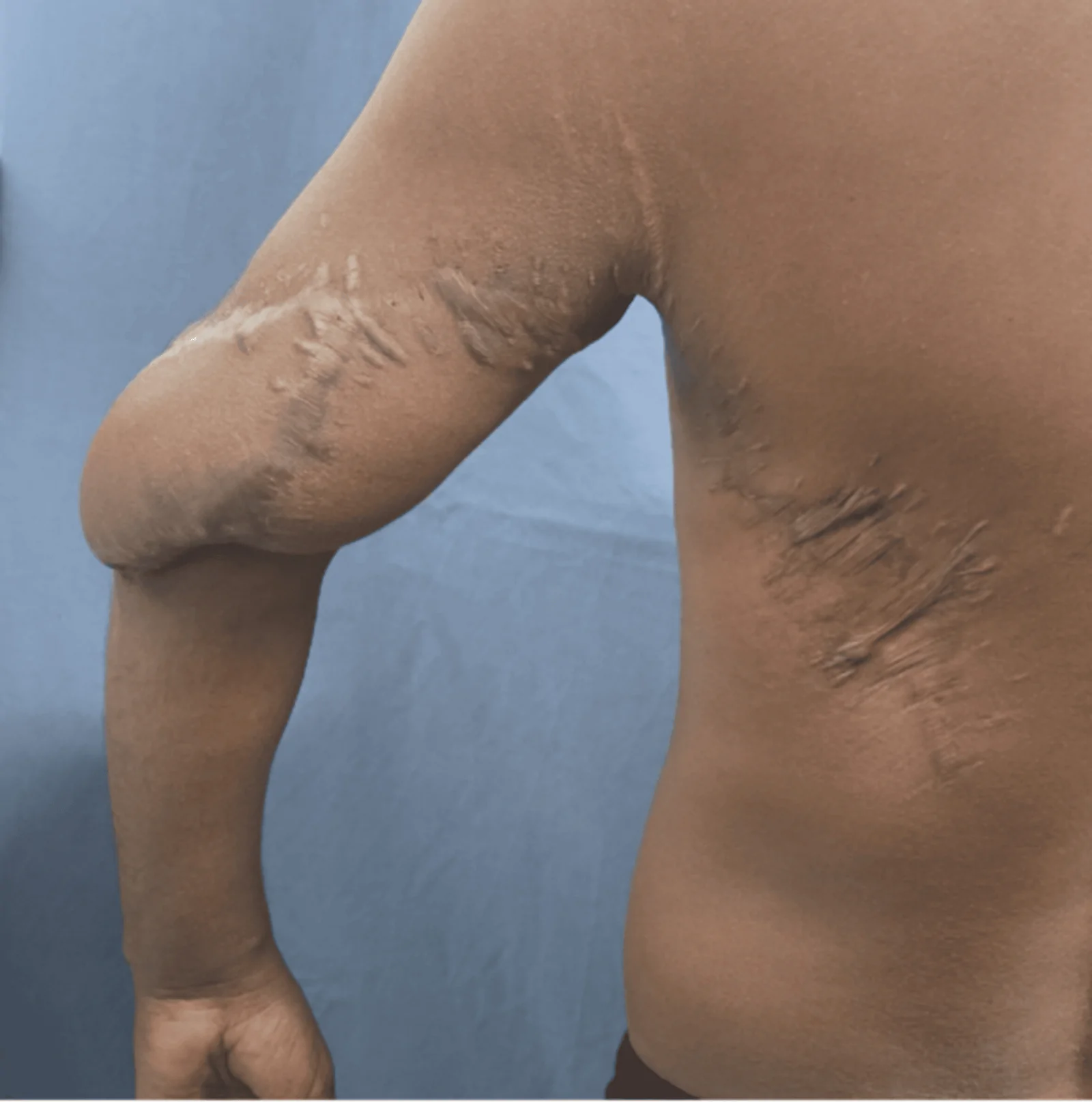
Follow-up clinical picture after one year

**Figure 9:**
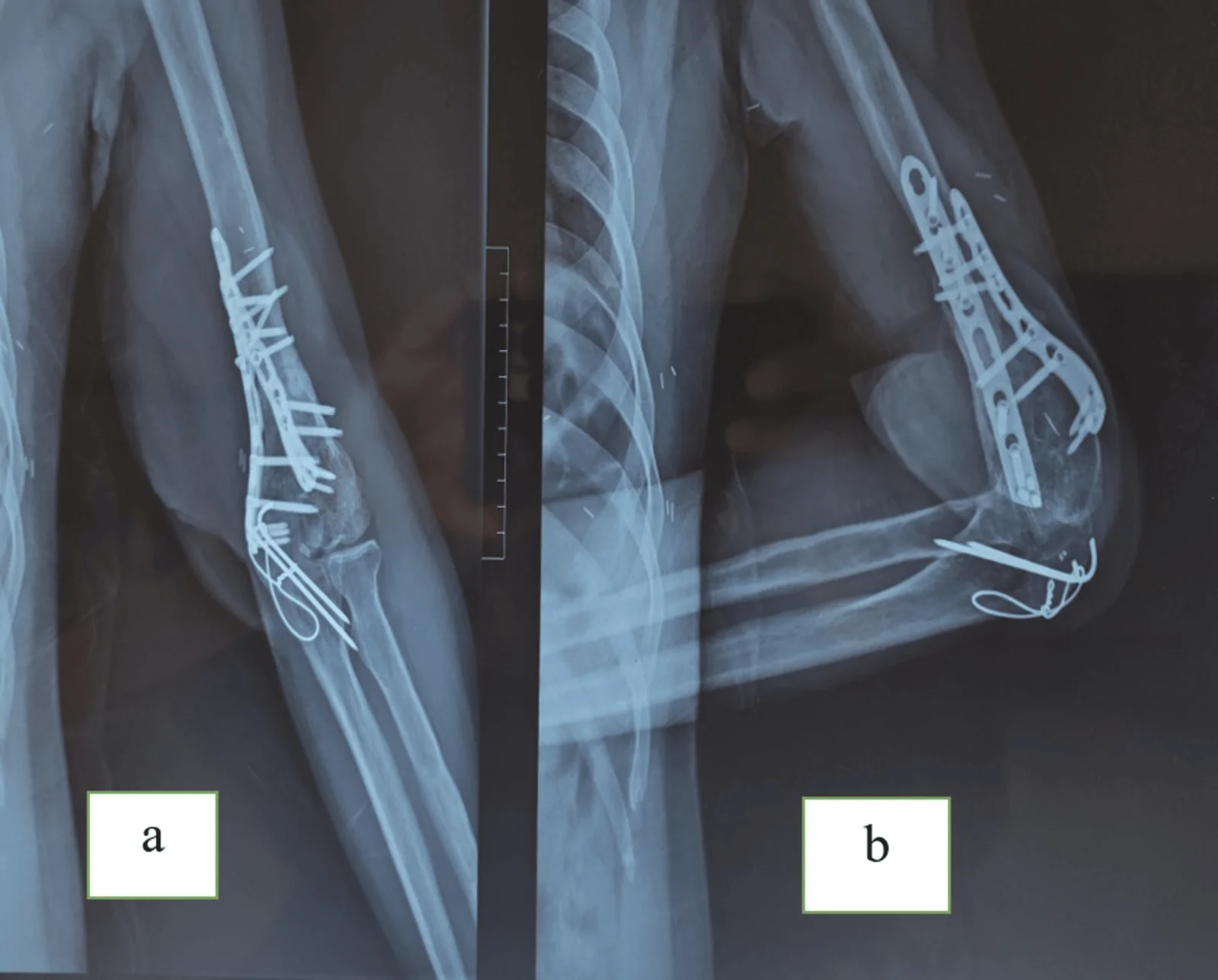
Follow-up X-ray after one year

At one-year follow-up, the patient demonstrated satisfactory wound healing with no clinical evidence of recurrent infection. Radiographs showed maintained alignment with progression toward fracture healing. The patient reported significant improvement in pain and was able to perform activities of daily living independently. Elbow range of motion also improved compared to the preoperative status, resulting in satisfactory functional recovery (Figure 8 and Figure 9). The final elbow range of motion was 130° of flexion.

## Discussion

Chronic post-traumatic infections around the elbow are difficult to manage because of the limited soft tissue envelope, subcutaneous location of the joint, and proximity to important neurovascular structures. Implant-associated infections following distal humerus fixation are frequently associated with persistent sinus discharge, stiffness, exposed hardware, and compromised local vascularity, all of which contribute to poor functional outcomes if not managed appropriately [4].

Successful treatment of chronic osteomyelitis requires a systematic approach aimed at eradication of infection, preservation of skeletal stability, and restoration of soft tissue coverage. Established principles include aggressive debridement, removal of infected implants, dead space management, culture-directed antibiotic therapy, fracture stabilization, and timely reconstruction [2,3]. In the present case, a staged limb salvage strategy was adopted to address each of these components sequentially. Initial implant removal and extensive debridement helped reduce the infective burden, while placement of antibiotic-impregnated cement beads and administration of culture-sensitive antibiotics improved the local wound environment prior to definitive reconstruction.

Soft tissue reconstruction remains a key component in the management of infected elbow defects. Vascularized muscle flaps improve tissue perfusion, help obliterate dead space, enhance local antibiotic delivery, and create a favorable environment for wound healing and infection control [5,6]. Among the available options, the pedicled latissimus dorsi flap continues to be considered a dependable workhorse flap for elbow reconstruction because of its reliable thoracodorsal blood supply, large muscle bulk, and wide arc of rotation [6-8]. In addition, pedicled transfer avoids the need for microvascular anastomosis, thereby reducing operative complexity in appropriately selected patients [7].

Chronic implant-associated distal humerus infections are particularly challenging because they are often accompanied by scarred soft tissues, retained hardware, stiffness, and poor local vascularity. In such situations, single-stage procedures may carry a higher risk of persistent infection or wound complications. The staged protocol employed in this case allowed adequate infection control before definitive fixation and flap coverage. Negative pressure wound therapy also served as a useful bridge by optimizing the wound bed prior to reconstruction. Reconstruction around the elbow remains challenging because of limited local soft tissue availability and the need to preserve functional mobility of the joint. Available reconstructive options include local rotational flaps, fasciocutaneous flaps, free tissue transfer, and muscle flaps. In the present case, the presence of chronic infection, scarred soft tissue, and exposed underlying structures favored the use of a pedicled latissimus dorsi muscle flap. The flap provided adequate tissue bulk, reliable vascularity, and durable coverage while avoiding the technical complexity associated with microvascular free tissue transfer.

Previous studies have highlighted the effectiveness of the pedicled latissimus dorsi flap in complex elbow defects. Al-Qattan reported satisfactory outcomes in severe traumatic soft tissue injuries around the elbow associated with peripheral nerve involvement [9]. Hur et al. also demonstrated reliable coverage and favorable flap survival in moderate-to-large elbow defects [8]. Similarly, Choudry et al. emphasized the importance of early and durable soft tissue coverage in preventing recurrent infection and preserving upper limb function [4].

In our patient, staged infection control followed by definitive skeletal stabilization and pedicled latissimus dorsi flap reconstruction resulted in durable wound healing without recurrence of infection at one-year follow-up. The flap provided stable vascularized coverage over the compromised elbow region and contributed to satisfactory functional recovery. This case highlights the importance of a multidisciplinary approach involving orthopedic and plastic surgical teams in achieving successful limb salvage in complex post-traumatic elbow infections.

Limitation(s) of the case report

This report is subject to the inherent limitations of a single case, precluding generalization of outcomes or statistical validation, and no comparison was made against alternative reconstructive options such as fasciocutaneous or free flaps. The one-year follow-up period, while informative, may not be sufficient to detect late complications, including recurrent infection, implant failure, or donor site morbidity such as shoulder weakness or contour deformity following latissimus dorsi harvest. Only a single organism, *Escherichia coli*, was isolated on culture; chronic osteomyelitis is often polymicrobial, and prior antibiotic exposure at other centers may have influenced culture yield. As with any single case report, the findings cannot be generalized across patients with differing age, comorbidities, or extent of bone and soft tissue loss.

## Conclusions

Chronic implant-associated infections following distal humerus fixation remain challenging because of persistent infection, compromised soft tissues, and associated functional limitation. Successful management requires meticulous staged treatment with aggressive debridement, infection control, skeletal stabilization, and timely soft tissue reconstruction. In our patient, the pedicled latissimus dorsi flap provided reliable vascularized coverage and contributed to durable wound healing without recurrence of infection at one-year follow-up. This case highlights the importance of a multidisciplinary limb salvage approach in achieving satisfactory functional and clinical outcomes in complex post-traumatic elbow infections.
